# Gender Differences in Trends in Incidence and Mortality of Acute Myocardial Infarction in the Small Island Developing State of Barbados

**DOI:** 10.7759/cureus.56729

**Published:** 2024-03-22

**Authors:** Arianne Harvey, Christina Howitt, Jacqueline M Campbell, Shelly-Ann A Forde, Ian Hambleton, Ivanna Bascombe, Simon G Anderson, Dawn Scantlebury, Rudolph Delice, Natasha P Sobers

**Affiliations:** 1 Faculty of Medical Sciences, The University of the West Indies, Bridgetown, BRB; 2 George Alleyne Chronic Disease Research Centre, Caribbean Institute for Health Research, The University of the West Indies, Bridgetown, BRB

**Keywords:** ischemic heart disease, cardiovascular disease, care pathway delays, incidence rate, mortality trends, non-communicable disease, gender differences, small island developing states, acute myocardial infarction

## Abstract

Objective

To determine trends, identify predictors of acute myocardial infarction (AMI) incidence and mortality, and explore performance metrics for AMI care in Barbados.

Methods

Data on all cases diagnosed with AMI were collected by the Barbados National Registry for Non-Communicable Diseases (BNR) from the island’s only tertiary hospital, the Queen Elizabeth Hospital, and the National Vital Registration Department. Participants who survived hospital admission were then followed up at 28 days and one year post event via telephone survey and retrieval of death certificates. Age-standardized incidence and mortality rates were calculated. Determinants of mortality at 28 days were examined in multivariable logistic regression models. Median and interquartile ranges (IQR) were calculated for performance metrics (e.g., time from pain onset to reperfusion).

Results

In a 10-year period between 2010 and 2019, 4,065 cases of myocardial infarction were recorded. The median age of the sample was 73 years (IQR: 61,83), and approximately half (47%) were female. Over a 10-year period, standardized incidence increased in women on average yearly by three per 100,000 (95% CI: 1 to 6; p=0.02), while in men, the average increase per year was six per 100,000 (95% CI: 4 to 8; p<0.001). There was no increase in 28-day mortality in women; mortality in men increased each year by 2.5 per 100,000 (95% CI: 0.4 to 4.5; p=0.02). The time from arrival at the hospital to the ECG was 44 minutes IQR (20,113).

Conclusion

AMI incidence and mortality are increasing in Barbados, and men have a higher velocity of mortality rate increase than women, which contradicts global data.

## Introduction

Acute myocardial infarction (AMI) is a common presentation of ischemic heart disease (IHD): the leading cause of death and disability worldwide, accounting for 9.1 million deaths in 2019 [1]. AMI incidence is increasing worldwide, but IHD mortality rates have exhibited a steady decline by almost a third from 1990-2019 [2]. There is sparse AMI incidence data available and unexplored mortality data for developing countries; thus, historically, IHD mortality reduction strategies in lower-income and developing countries have been led by insights from data from high-income, developed countries [3,4].

Recent studies have begun to present country-level IHD evidence for the mid- and lower-income regions, improving the availability of data and highlighting the variability in mortality rate reduction across individual countries associated with gross national income. For example, in contrast to a significant reduction in high-income North America, mortality rates in Central America, Mexico, and the Latin Caribbean remained broadly unchanged [5]. AMI incidence and mortality characteristics can illustrate IHD epidemiology [6]. Research that better elucidates these for developing countries in detail, currently not available to global analytics teams, is needed to inform country-level health strategy [2,5].

The purpose of this study was to describe national AMI incidence and mortality trends in Barbados, an English-speaking, Caribbean, high-income, Small Island Developing State (SIDS) with a majority Black population, between 2010 and 2019.

We hypothesized that AMI incidence and mortality would be greater than in larger high-income countries but comparable to low- and middle-income countries in the region because of the risk of contributing to the heavy cardiovascular disease (CVD) burden. We expected to observe delays in access to AMI treatment pathways comparable to other developing states that might contribute to mortality [7-10]. The aims were to (1) describe the age-standardized incidence and mortality rates by gender; (2) determine the relationship between AMI 28-day case fatality rate and prevalent CVD risk factors; and (3) describe delays along the local care pathway to AMI treatment.

## Materials and methods

Study design

Data for this study were collected prospectively by the Barbados National Registry for Non-communicable Diseases (BNR). The BNR is a multi-disease registry that collects data on all cases of myocardial infarctions in Barbados [11]. Cases are found largely through active surveillance of private and public facility records, with minimal data collected through referrals from healthcare providers. Cases are followed up to determine vital status at 28 days and one year using the National Vital Registration Department of the Barbados Government (NVRD), which registers all deaths on the island.

Settings

Barbados is a high-income SIDS of 166 square miles, has a population of 278,300 (2022) [12], and is located in the Southern Caribbean. Healthcare is available for free at the point of delivery of all government-owned facilities, which include a single public tertiary care hospital (Queen Elizabeth Hospital, QEH) and nine primary care centers (also known as polyclinics). Additionally, there are private sector facilities, including a 30-bed hospital facility, four major urgent care centers, and hundreds of smaller primary clinics that offer various services for a fee. At the time of this study, only the government-owned public hospital offered reperfusion using fibrinolytics for ST-elevation myocardial infarctions (STEMI), and timely primary percutaneous coronary intervention was not available. Because of its reperfusion capacity, the government-owned hospital was the default treatment site for those with AMI.

Participants

To answer our research questions, the data analytics required two related but distinct datasets. Incidence data were obtained from the BNR prospective data collection, and mortality data were obtained from the NVRD. The patients in the BNR dataset included all Barbadian citizens and permanent residents with a diagnosis of AMI observed at public and private hospitals and primary care clinics in Barbados. Patients are considered to have had an AMI if that diagnosis is stated in the initial and final diagnosis sections of the charts. In Barbados, clinicians make the diagnosis of AMI based on the current universal and epidemiological definitions [6]. Patients are often registered at admission and followed until discharge. Discharge notes are, additionally, assessed for missed AMI cases or those that occurred or were diagnosed post admission.

Data on the event and in hospital care, where applicable, were entered into the BNR database. A registered death will not always be in the BNR prospective incidence database; thus, the NVRD was used to identify the vital status of registered patients. Additionally, we also included as incident cases those whose cause of death from the NVRD was registered as AMI or IHD during the study period. Using only those participants registered from the BNR incident dataset, persons who survived hospital admission were followed up at 28 days and again at one-year post event via telephone survey and using death certificates. Patients must have resided in Barbados for at least one year before the event to have been eligible for the study.

Data collection and variables

**Figure 1:**
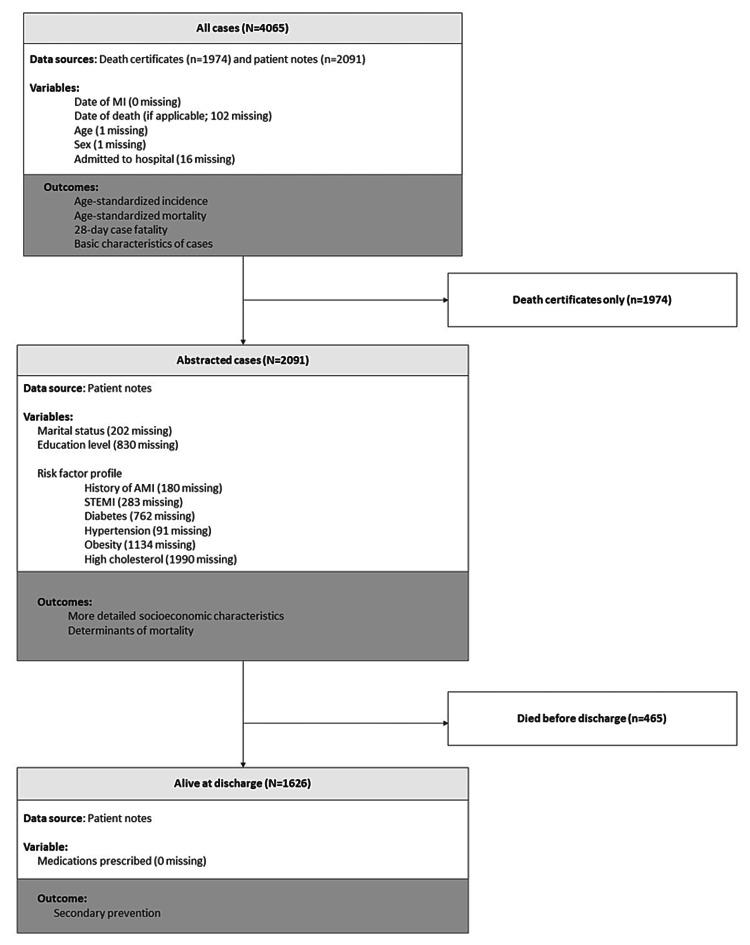
Flowchart showing data sources, variables used, and outcomes for this analysis

The data sources used in this analysis, along with the associated outcomes and summaries of missing data points, are shown in Figure 1. Dates of myocardial infarction (MI) and death (if applicable), age, sex, and whether the case was admitted to the hospital were either abstracted from patient notes if possible (n=2,091) or from death certificates (n=1,974). This information was used to calculate age-standardized incidence and mortality and 28-day case fatality and to provide basic characteristics of the cases. For hospitalized cases, more detailed characteristics were available (marital status, education, and whether they had a first-degree relative with a history of AMI). To determine the factors associated with 28-day case fatality in this population, based on a priori hypothesis from previous studies, the clinical variables abstracted include a history of diabetes mellitus, hypertension, hypercholesterolemia, obesity, and past medical history of AMI. To assess delays along the care pathway, the following times were collected: onset of chest pain/first symptom; arrival at hospital; first ECG; and reperfusion. For those who were alive at discharge (n=1,626), the medications prescribed for secondary prevention were obtained.

Statistical methods

Descriptive statistics are presented either as percentages of cases with 95% CIs (CI) (most sociodemographic and clinical characteristics) or medians with interquartile range (age, time to diagnostics and treatment). To account for age differences between female and male cases, and to compare the sociodemographic characteristics of cases with the Barbadian population according to the 2010 census, prevalence rates were directly standardized using the 2010 Barbados population [13]. The UN World Population Prospects 2022 population estimates for Barbados from 2010 to 2019 were used to provide the denominators for crude and age- and sex-specific rates of incidence and mortality [14]. Age-specific incidences were calculated for nine age groups (e.g., 0-9, 10-19, 20-29, 80 years and over). The rates were also directly standardized to the age distribution of the WHO 2000-2025 Standard Population. CIs for these rates were computed based on the gamma distribution using a modified formula [15]. This method is recommended for small populations as it produces valid CIs even when the number of cases is small.

For the plots of incidence and mortality over time, to better visualize the trend, a smoothed line was computed using a robust locally weighted regression [16]. A linear regression model with time as a predictor was fitted to test for a trend.

Determinants of case fatality at 28 days were examined in multivariable logistic regression models, with the results presented as odds ratios (OR) and 95% CIs. This model type was selected rather than a survival model that allows for censoring because very few participants were lost to follow-up, with only 13 out of 2,091 cases missing vital status at 28 days. Potential determinants included traditional risk factors (diabetes, hypertension, obesity) and medical factors (previous AMI, aspirin use). These were selected based on a priori knowledge and data availability. High cholesterol, which is an important risk factor for AMI, could not be included, which we acknowledge as a limitation in the data. The effect of each potential determinant on 28-day case fatality was first examined in men and women separately, with models adjusted for age, and then in models adjusted for age along with the other determinants in the same category (either traditional risk factors or medical factors). We consider a P value of <0.05 as statistically significant.

## Results

AMI incidence

The age- and sex-specific incidence of AMI in Barbados is presented in Table 1. In women, incidence ranged from 6.1 (95% CI: 0.2, 33.7) per 100,000 in the 0-9-year age group to a peak of 12,141.7 (95% CI: 11,356.1, 12,967.2) per 100,000 people in the 80 years and over age group. In men, the incidence ranged from 23.5 (95% CI: 6.4, 60.2) per 100,000 people in the 0-9-year age group to a peak of 11,260.3 (95% CI: 10,330.5, 12,251.4) per 100,000 people in the 80 years and over age group. Incidence standardized to the WHO 2000-2025 population was significantly higher in men 1,037.5 (95% CI: 993.0, 1,083.7) per 100,000 than women 639.6 (95% CI: 609.1, 671.5) per 100,000.

**Table 1:** Age-specific and age-standardized incidence of acute myocardial infarction in Barbados per 100,000 people (2010–2019)

			Females		Males			
Age group	Cases	Population	Incidence	95% CI	Cases	Population	Incidence	95% CI
0-9	1	16,512	6.1	(0.2, 33.7)	4	17,008	23.5	(6.4, 60.2)
10-19	1	18,552	5.4	(0.1, 30)	3	19,292	15.6	(3.2, 45.5)
20-29	2	18,750	10.7	(1.3, 38.5)	12	18,626	64.4	(33.3, 112.5)
30-39	28	19,742	141.8	(94.3, 205)	57	18,764	303.8	(230.1, 393.6)
40-49	82	21,039	389.8	(310, 483.8)	165	19,520	845.3	(721.2, 984.6)
50-59	183	21,039	869.8	(748.3, 1,005.4)	371	18,566	1,998.3	(1,800.1, 2,212.4)
60-69	312	15,323	2,036.2	(1,816.5, 2,275.1)	519	13,499	3,844.6	(3,520.9, 4,190)
70-79	407	9,730	4,183.1	(3,786.5, 4,609.9)	488	7,583	6,435.6	(5,877.2, 7,032.7)
80+	888	7,314	12,141.7	(11,356.1, 12,967.2)	540	4,796	11,260.3	(10,330.5, 12,251.4)
Age-standardized rate (world)			639.6	(609.1, 671.5)			1,037.5	(993.0, 1,083.7)

**Figure 2:**
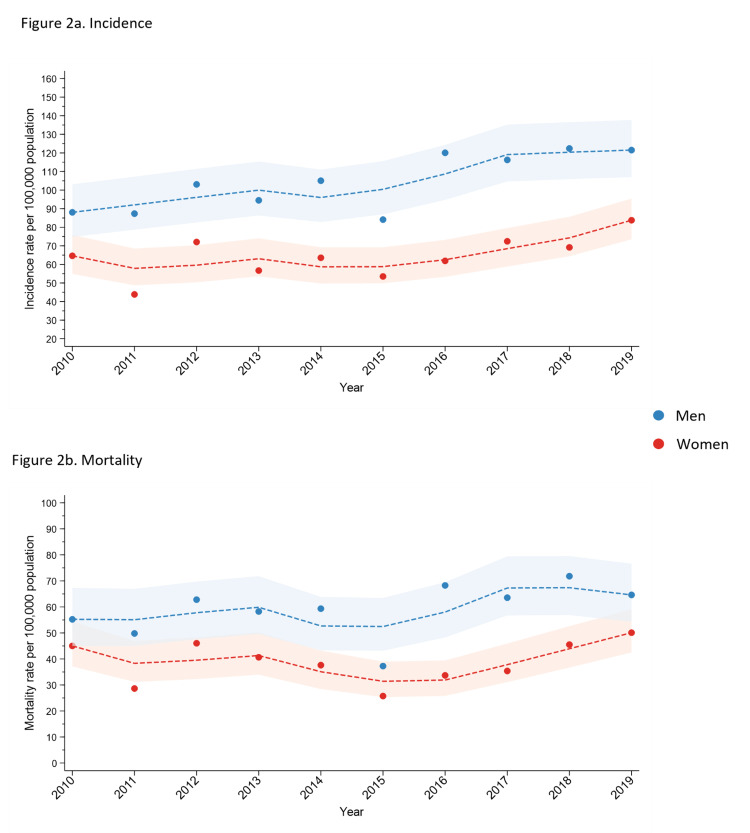
Trends in age-standardized myocardial infarction incidence and mortality rates by sex and year in Barbados (2010–2019)

Trends in age-standardized incidence are shown in Figure 2A. Over the 10-year period, age-standardized incidence did not increase in women but did increase in men with an average annual increase of four cases per 100,000 people (95% CI: 2-6; p<0.001).

Characteristics of cases

The characteristics of the AMI cases registered between 2010 and 2019 are shown in Table 2. Approximately half of the sample was female (47%). The median age was 73 years (IQR: 61, 83), with women on average being older than men (median of 78 years compared with 69 years). According to the crude prevalence estimates, the socioeconomic profile of cases differed by gender in marital status and educational attainment. Women were more likely to be widowed (19% compared with 5% in men) and less likely to be married (25% compared with 42% in men). Male cases had higher education levels, with 32% completing secondary school compared to 24% in women, and 15% completing college or university compared to 8% of women. However, these differences were attributable to the older age of female cases, as the age-standardized estimates are similar in men and women. After standardizing to the 2010 Barbadian population, 25% and 12% of cases had attained a secondary school and tertiary-level education, respectively. The proportion of cases with a tertiary-level education is similar to national estimates (14%). Conversely, the proportion with a secondary school education was lower than the national average (67%). It should be noted that this comparison should be made with caution owing to the large amount of missing data points for education (missing for 830 or 40% of cases).

**Table 2:** Sociodemographic and clinical characteristics of MI cases in Barbados (2010–2019) MI: myocardial infarction; STEMI: ST-elevation myocardial infarctions; 1: values in this case are median (interquartile range)

	Crude prevalence (95% CI)			Standardized prevalence (95% CI)		
For all cases (n=4,065)	Women	Men	Total	Women	Men	Total
	(n=1,905)	(n=2,159)		(n=1,905)	(n=2,159)	
Age in years^1^	78 (66, 86)	69 (58, 80)	73 (61, 83)	-	-	-
Admitted to hospital	58.8 (56.6, 61.0)	60.9 (58.8, 62.9)	59.9 (58.4, 61.4)	-	-	-
For abstracted cases only (n=2,061)						
Marital status						
Single	37.3 (34.3, 40.4)	37.5 (34.7, 40.4)	37.4 (35.3, 39.5)	30.3 (24.3, 52.9)	44.0 (32.4, 66.6)	41.6 (34.5, 51.5)
Married	24.9 (22.3, 27.8)	41.7 (38.9, 44.6)	34.1 (32.1, 36.2)	17.0 (12.9, 40.3)	21.9 (19.2, 39.0)	19.3 (17.6, 25.2)
Divorced or separated	8.0 (6.4, 9.9)	6.7 (5.4, 8.3)	7.3 (6.2, 8.5)	5.1 (3.1, 30.1)	3.1 (2.0, 23.1)	3.8 (3.1, 10.2)
Widowed	19.3 (16.9, 21.9)	5.1 (4.0, 6.5)	11.6 (10.3, 13.0)	4.2 (3.6, 28.3)	1.2 (0.9, 23.2)	2.6 (2.4, 9.2)
Education						
Less than secondary school	27.0 (24.3, 30.0)	14.3 (12.3, 16.4)	20.1 (18.4, 21.9)	7.8 (6.4, 31.6)	9.0 (4.5, 29.0)	9.4 (6.5, 16.5)
Secondary school completed	23.9 (21.3, 26.7)	32.2 (29.6, 35.0)	28.5 (26.6, 30.4)	23.6 (18.3, 46.2)	25.6 (19.7, 44.6)	25.2 (21.6, 32.2)
College/university completed	7.7 (6.1, 9.5)	15.2 (13.3, 17.4)	11.8 (10.5, 13.2)	7.0 (4.4, 31.0)	13.7 (8.4, 33.3)	11.7 (8.5, 18.8)
Clinical and risk factor characteristics						
History of MI	24.5 (21.9, 27.4)	22.2 (19.9, 24.7)	23.2 (21.5, 25.1)	14.2 (10.4, 36.7)	15.8 (10.4, 35.5)	15.8 (12.5, 22.8)
STEMI	32.6 (29.7, 35.6)	44.6 (41.8, 47.5)	39.1 (37.0, 41.2)	22.8 (17.9, 45.6)	37.1 (28.0, 57.5)	33.4 (27.9, 42.0)
Diabetes	56.9 (53.8, 60.0)	37.2 (34.5, 40.1)	46.2 (44.1, 48.4)	28.7 (24.2, 51.0)	18.1 (15.7, 35.8)	22.1 (20.5, 27.9)
Hypertension	79.9 (77.2, 82.3)	62.9 (60.1, 65.7)	70.7 (68.7, 72.6)	40.5 (34.9, 62.9)	36.5 (30.3, 54.8)	38.7 (35.0, 45.7)
Obese	40.0 (37.0, 43.2)	28.1 (25.5, 30.8)	33.5 (31.5, 35.6)	27.8 (22.1, 50.6)	29.9 (19.8, 51.7)	31.3 (25.1, 40.5)
High cholesterol	3.0 (2.1, 4.3)	2.6 (1.9, 3.8)	2.8 (2.2, 3.6)	1.2 (0.6, 27.9)	1.3 (0.8, 22.2)	2.8 (1.0, 8.3)

Approximately 60% of cases were admitted to hospital. The proportion with a previous AMI was similar in men vs women (23%), but the proportion classified as ST-elevation myocardial infarctions (STEMI) was higher in men (45% vs 33% in women). After standardizing for age, the difference in STEMI prevalence persisted in the prevalence estimates but lost statistical significance: 37.1 (95% CI: 28.0, 57.5) in men compared with 22.8 (95% CI: 17.9, 45.6) in women. Female cases had a higher prevalence of diabetes (57% vs 37%), hypertension (80% vs 63%), and obesity (40% vs 28%) than their male counterparts. However, these differences were attenuated in the age-standardized estimates, indicating that they are attributable to the age difference by gender of the cases.

AMI mortality

Age-standardized mortality in the 10-year period between 2010 and 2019 was 386 per 100,000 people (95% CI: 363, 410) in women and 587 (95% CI: 554, 621) in men. Changes in age-standardized mortality over time are shown in Figure 2b. Over the 10-year study period, there was no change in mortality in women or men, with the average annual change being 1.51 (95% CI: -0.87, 3.89) in men and 0.47 (95% CI: -1.66, 2.60) in women.

Determinants of vital status at 28 days

The 28-day case fatality rate for those who were hospitalized was 44.0%: 47.3% in women and 41.1% in men (P value for difference=0.007). The results of the regression analysis to examine determinants of the 28-day case fatality rate are shown in Table 3. In women, after adjusting for age, a previous diagnosis of diabetes was associated with higher odds of mortality at 28 days, with an OR (95% CI) of 1.89 (1.11, 3.21). However, in the model that includes all other potential predictors as well as age, this association is diminished and loses statistical significance (OR: 0.68; 95% CI: 0.23, 1.95). In men, after adjusting for age, both diabetes and hypertension were associated with increased odds of mortality at 28 days, with ORs (95% CI) of 4.06 (2.46, 6.73) and 2.97 (1.62, 5.44), respectively. However, after controlling for age and all other potential predictors, only the association between diabetes persists: OR: 3.81 (95% CI: 1.05, 13.91). In age-adjusted models, the acute use of aspirin (both on its own and in combination with prior use) was associated with a lower 28-day case fatality rate in both sexes. For acute use only, ORs (95% CIs) were 0.21 (0.14, 0.33) in women and 0.17 (0.11, 0.25) in men. For prior use and acute use, ORs (95% CIs) were 0.18 (0.10, 0.30) in women and 0.16 (0.09, 0.29) in men. When all other potential predictors were included in the model, there was no longer an association for either sex: for acute use only, ORs (95% CIs) were 0.38 (0.10, 1.35) in women and 0.43 (0.12, 1.52) in men. For prior use and acute use, ORs (95% CIs) were 0.46 (0.12, 2.16) in women and 0.56 (0.14, 2.16) in men.

**Table 3:** Determinants of 28-day mortality in MI cases in Barbados (2010–2019) MI: myocardial infarction; Model 1 is adjusted for age only; Model 2 includes all other predictors

	Model 1		Model 2	
	Odds ratio	95% CI	Odds ratio	95% CI
Women				
Diabetes	1.89	(1.11, 3.21)	0.68	(0.23, 1.95)
Hypertension	1.00	(0.56, 1.80)	0.49	(0.14, 1.66)
Obesity	1.15	(0.66, 2.00)	1.21	(0.44, 3.31)
Previous MI	1.15	(0.78, 1.70)	1.30	(0.57, 2.97)
Aspirin use (no recorded use is the reference category)	-	-	-	-
Prior use only	0.61	(0.34, 1.11)	0.46	(0.06, 3.50)
Acute use only	0.21	(0.14, 0.33)	0.38	(0.10, 1.35)
Prior use and acute use	0.18	(0.10, 0.30)	0.46	(0.12, 2.16)
Men				
Diabetes	4.06	(2.46, 6.73)	3.81	(1.05, 13.91)
Hypertension	2.97	(1.62, 5.44)	1.19	(0.20, 7.03)
Obesity	1.73	(0.98, 3.07)	2.88	(0.90, 9.23)
Previous MI	1.15	(0.75, 1.75)	1.48	(0.54, 4.05)
Aspirin use (no recorded use is the reference category)	-	-	-	-
Prior use only	0.76	(0.43, 1.33)	1.11	(0.16, 7.88)
Acute use only	0.17	(0.11, 0.25)	0.43	(0.12, 1.52)
Prior use and acute use	0.16	(0.09, 0.29)	0.56	(0.14, 2.16)

Assessment of secondary prevention medication

Aspirin or clopidogrel were the most prescribed secondary prevention medications, and the proportion of cases prescribed these medications was similar in women and men (84% and 80%, respectively; P value for difference: 0.134). Statins were prescribed for 71% of female cases and 67% of male cases (p=0.249). Beta-blockers were more commonly prescribed in women compared with men, at 69% and 62%, respectively (p=0.005). 

Delays along the local care pathway to AMI treatment

We calculated the average time between key points along the care pathway to AMI treatment to determine where delays occurred and whether there were differences by gender (Table 4). On average, there was a longer time between the onset of AMI and calling an ambulance for men compared with women, with a median (IQR) of 186 (73, 324) minutes in men and 119 (53, 351) minutes in women. Conversely, there was a longer time between AMI onset and reperfusion in women than in men, at 344 (230, 757) and 280 (215, 466) minutes, respectively. For all other metrics (time from onset of AMI to calling an ambulance, time from calling an ambulance to arrival at the hospital, time from arrival at the hospital to ECG, and time from the ECG to reperfusion), reflected as cumulative time from onset of AMI to reperfusion, the average time was within 10 minutes for both women and men. Median (IQR) door-to-ECG time (i.e., time from arrival at the hospital to ECG) was 44 (20, 113) minutes. For the 151 cases that were reperfused, the time from ECG to reperfusion was 82 (36, 154) minutes.

**Table 4:** Delays along the local care pathway to AMI treatment for hospitalized MI cases in Barbados (2016–2019) AMI: acute myocardial infarction; MI: myocardial infarction

	Women, median (IQR) in minutes	Men, median (IQR) in minutes	Total	P value for the mean difference between men and women
Time from onset of AMI to calling an ambulance	119 (53, 351)	186 (73, 324)	155 (32, 335)	0.363
Time from calling an ambulance to arrival at hospital	51 (42, 60)	49 (38, 61)	50 (38, 60)	0.220
Time from arrival at a hospital to ECG	51 (20, 124)	40 (20, 104)	44 (20, 113)	0.997
Time from the ECG to reperfusion	79 (51, 201)	87 (35, 145)	82 (36, 154)	0.630
Time from onset of AMI to reperfusion	344 (230, 757)	280 (215, 466)	295 (223, 522)	0.496

## Discussion

This paper presents, for the first time, 10 years of data from a national, prospective AMI registry in the high-income SIDS of Barbados. The data highlight key similarities and differences between local and international trends and provide insights into the potential avenues for reducing disease burden in this setting.

This study found a higher incidence of AMI in men, consistent with international data [17,18] and a trend toward increasing incidence in men. Moreover, men had a higher AMI mortality than women, and their mortality rates are increasing when compared to mortality in women.

Despite women having lower overall AMI mortality, case fatality rates for women were still found to be higher than for men in this population. Increasingly, more Barbadian men are dying from AMI than women, but the likelihood of dying from their individual AMI presentation is higher for a Barbadian woman than for a similarly presenting man. This aligns with the international data, which attributes this to women being older with a high comorbidity burden at AMI presentation and being less likely to receive guideline-directed therapy [19-21]. Sobers et al. have already shown gender-based differences in AMI management in the Barbadian setting [22]. Further research is needed to explore sex-specific differences in the characteristics and mortality of AMI presentations, particularly to tailor risk reduction strategies for AMI in Barbadian men, to explore factors that underpin AMI care biases against women, and to address the inequity of care.

In Barbados, men have lower rates of obesity and diabetes than women, report more physical activity, smoke more, and have similar rates of hypertension [23]. Their overall CVD risk factor burden is, therefore, likely less or the same as women. These nuanced differences in AMI gender data highlighted by our study allude to underrecognized or underexplored risk factors impacting AMI mortality in men specifically. One consideration is the role of behavioral risk factors (e.g., disparities in risk factor awareness and poor health-seeking behaviors), resulting in disparity in risk factor management.

Age-standardized mortality from AMI has remained unchanged over the 10-year period of 2010-2019, in contrast to worldwide trends that show a decrease in AMI mortality [24]. One potential explanation for the divergence of our data from global trends is the high and increasing burden of noncommunicable disease (NCD) risk factors in this population. From 2009 to 2019, the top risk factors [25] driving mortality in Barbados (i.e., high fasting glucose, increased body mass index, elevated blood pressure, dietary risk, and chronic kidney disease) have increased by about 30%, and IHD remains the leading cause of death in Barbadians. Sobers et al. demonstrated that rates of improvement in AMI mortality in Barbados because of improved treatment of AMI were hindered by the increasing impact of diabetes and obesity in the population [8]. This supports the use of national public health strategies to aggressively target risk factor prevention and treatment, such as sugar taxation, healthy food labeling, public education on NCDs, health provider education, and national investment in risk factor surveillance and research.

The data identified type 2 diabetes mellitus as the strongest and only significant predictor for increased AMI mortality in our population. Additionally, diabetic men had a higher mortality than women. This differs from studies in other settings that have identified multiple predictors of AMI mortality [26].

Diabetes mellitus as the strongest predictor of AMI mortality in Barbados, particularly for men, may be layered. Documentation of diabetes mellitus versus complications of diabetes as a comorbid state to AMI presentations may need to be discerned to elucidate the true severity of impact on AMI survival. Overweight and obesity are often comorbid with diabetes mellitus but are themselves independent predictors of increased AMI mortality [27]. The underrecognition and underreporting of overweight and obesity may cause the attribution of increased AMI mortality to the recorded diabetes diagnosis.

Finally, this study explored time delays along the pathways of care experienced by Barbadian patients presenting with AMI. Delays in access to care, particularly access to definitive revascularization for ST-elevation myocardial infarction (STEMI), are associated with poor outcomes and increased AMI mortality in developing countries [10,28]. This study highlighted delays at multiple steps in accessing care for AMI patients within the country’s healthcare system. These findings parallel the characteristics of AMI processes of care documented for other developing countries [10,28-30]. Barbados does not have an established protocol for definitive STEMI management; hence, delays in time to reperfusion observed in this study were expected. These delays may play a role in mortality rates observed in this population and warrant further research.

Limitations

This study may have been affected by both information bias and selection bias. The most likely form of selection bias to have affected this study was lost to follow-up because some patients were not contactable post discharge. This may have been caused by a change of address or contact numbers. It is expected that patients who died post discharge would have been registered on assessment of the NVRD’s records. Numerous possible sources of information bias are identified. Patient records at the QEH are largely handwritten, and it is not uncommon to find those that are difficult to interpret, resulting in the possibility of misread words or numerals. This is minimized by the ability to include photos within the database and the provision made for communication with Internal Medicine specialists to clarify records of concern. Additionally, records may be difficult to retrieve or irretrievable. This is especially so for death certificates within the hospital. Records are retrieved by QEH records department staff, and three attempts must be made to obtain a record before it is considered irretrievable. Additionally, the discussion on STEMI and the impact of evolution in reperfusion availability access on mortality trends was limited because of the small dataset available, which was not powered to detect changes in STEMI over time. More accurate data collection done prospectively could address this limitation for future analyses.

## Conclusions

Similar to many other countries, Barbados's epidemiological transition has been rapid. This has resulted in a high-income status that has afforded a diminished role for physical labor and enhanced access to ultraprocessed foods, generating an increasingly at-risk population for AMI. This, juxtaposed with the public health limitations of SIDS status (i.e., poor health education, socioeconomic and cultural barriers to healthcare reform, inadequate systems of care, and lack of a health policy), has created a crucible for worsening AMI incidence and mortality. The greater mortality impact of this combined risk on Barbadian men suggests that additional factors, such as health awareness and healthcare utilization, may modify the natural history of disease and eventual outcomes. We recommend data-driven tailored solutions toward public education of AMI risk awareness and addressing barriers to healthcare utilization for Barbadian men.

The demonstration of the disparity in the treatment outcomes of Barbadian women presenting with AMI and performance data shows that Barbados has similar challenges in AMI care access, as described worldwide, and has implications for implementation for outcome improvement. We recommend public education on the recognition of AMI symptoms and urge the search and development of protocols that facilitate timely navigation from prehospital assessment to definitive treatment. This should be aimed toward closing gender gaps in equitable care for AMI and eliminating delays along the AMI systems of the care pathway, with endorsement from government policy and funding.
